# Giant recurrent liposarcoma of the retroperitoneum – A surgical challenge: A case report

**DOI:** 10.1016/j.ijscr.2020.11.051

**Published:** 2020-11-12

**Authors:** Zied Mahjoubi, Walid Zakhama, Aymen Sakly, Manel Njima, Aymen Mnasser, Yassine Binous

**Affiliations:** a Department of Urology, Hospital Taher Sfar, 5100, Mahdia, Tunisia; b Department of Pathology, Hospital Fattouma Bourguiba, Farhat Hached, 5000, Monastir, Tunisia

**Keywords:** Dedifferentiated liposarcoma, Retroperitoneum, Surgical resection, Recurrence, Case report

## Abstract

**Introduction:**

Sarcomas are a rare entity with heterogeneous clinical behavior. We herein present the case of a giant retroperitoneal dedifferentiated liposarcoma and aim to remind the clinical, histological, and therapeutic features of this rare tumor.

**Presentation of case:**

An 80-year-old patient was complaining of significant abdominoscrotal swelling. On physical examination, a 25-cm swelling extending from the right iliac fossa to scrotums, was observed. CT scan revealed a retroperitoneal fatty mass with necrotic areas. Ultrasonography-guided biopsy was carried. Pathological report demonstrated a dedifferentiated sarcoma. An en bloc resection of the tumor was performed through a right iliac incision extended to the scrotum. After one year of follow up, the patient had a 30-cm local recurrence. After complete resection, the patient died two days after the procedure due to neurological distress.

**Discussion:**

‘Giant’ liposarcomas over 20kg are extremely rare. CT-Scan and MRI are very useful for defining their size and limits. The resection of a retroperitoneal sarcoma of remarkable size is a challenge for the surgeon owing to the anatomical site that makes it hard to obtain safe margin and to the adherences with the contiguous organs. Dedifferentiated histologic subtypes and negative surgical margins are associated with poor prognosis. This explains the high rate of local recurrence after surgical excision.

**Conclusion:**

In rare cases, retroperitoneal dedifferentiated liposarcomas can extend through the inguinal canal to the scrotum. Surgical resection obtaining negative margins, remains the curative treatment that reduces the risk of recurrence. Careful follow-up to detect early recurrence is essential for optimal care.

## Highlights

• Giant retroperitoneal dedifferentiated liposarcoma can extend to the scrotum.
• Dedifferentiated histologic subtypes of liposarcomas are associated with poor prognosis.
• Performing an en bloc resection through a right iliac incision extended to the scrotum.
• Obtaining negative margins after surgical resection is the curative treatment.
• Careful follow-up to detect early recurrence is essential for optimal care.

## 1 Introduction

Sarcomas are a rare entity arising from the mesoderm with heterogeneous clinical behavior. The genitourinary tract location represents only 5%. Approximately 12–15 % of soft tissue sarcomas are arising from the retroperitoneum. Among histologic subtypes, liposarcoma is the most common (30–50 %) [1]. A wide surgical resection remains the main treatment. We herein present the case of a giant retroperitoneal dedifferentiated liposarcoma and aim to remind the clinical, histological, and therapeutic features of this rare tumor. This work has been reported in line with the SCARE 2018 criteria [2].

## 2 Presentation of case

An 80-year-old patient was referred to our emergency department complaining of asthenia, vomiting, and significant abdominoscrotal swelling. He had no history of chronic disease or surgery. The initial swelling had started in the scrotum two years earlier following a benign scrotal trauma and thereafter abdominal distention was recognized and increasing gradually but neglected by the patient.

**Fig. 1:**
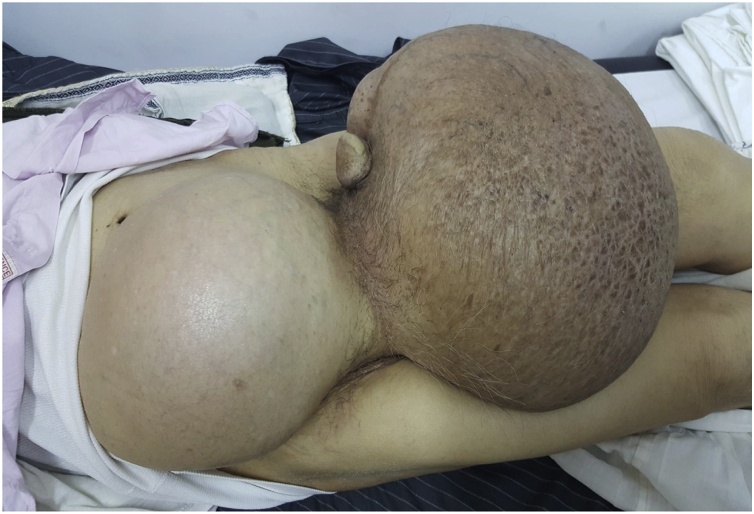
An enlarged painful swelling with ill-defined margins measuring 25 cm extending from the right iliac fossa to scrotums.

On physical examination, the patient showed a severe nutritional deficiency with muscle and adipose tissue loss. The upper extremities and the face demonstrated cachexia, and the abdomen was severely distended, owing to an enlarged painful swelling with ill-defined margins measuring 25 cm extending from the right iliac fossa to scrotums. The diameter of the scrotal tumor was 50 cm with edematous skin, and the penis had sunk into the mass (Fig. 1). Slight bilateral foot edema was present and mild dyspnea was evident.

Laboratory investigations revealed a severe hypochromic and microcytic anemia (Hb 8,3 g/dcl), and a high serum level of lactic dehydrogenase (550 U/L).

**Fig. 2:**
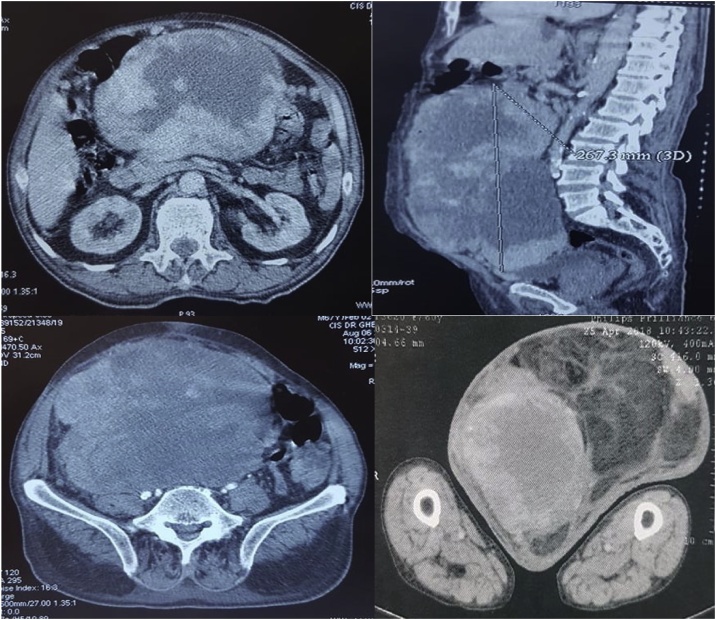
CT scan images revealing a heterogeneously enhancing fatty mass with solid nodules and necrotic areas, with craniocaudal extension of over 40 cm from the retroperitoneum to the inguinoscrotal region, compressing the abdominal aorta and the intestines to the left side of the abdomen.

The CT scan revealed a heterogeneously enhancing fatty mass with solid nodules and necrotic areas, with craniocaudal extension of over 40 cm from the retroperitoneum to the inguinoscrotal region, compressing the abdominal aorta and the intestines to the left side of the abdomen. The testicles were hypo trophic and repressed. No local infiltration or distant metastasis was detected (Fig. 2). An ultrasonography-guided biopsy was carried. After the immunohistochemical study, the pathological report demonstrated a grade II FNCLCC dedifferentiated sarcoma.

The case was presented at a multi-disciplinary team meeting including the urologists, general surgeons, pathologists and radiologists. Surgical management option was selected by the multidisciplinary team basing on the tumor resectability assessment and the ability of the patient to tolerate the surgery. The procedure was promptly planned and an en bloc resection of the tumor was performed through a right iliac incision extended to the scrotum. The main difficulty was to guarantee clear margins sparing major vessels or adjacent organs.

**Fig. 3:**
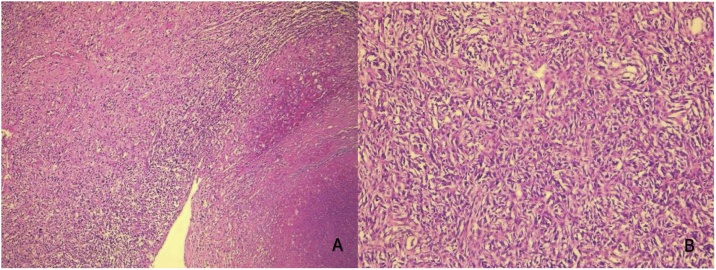
Undifferentiated areas with spindle cells arranged in storiform bundles (A: HEx40, B: HEx200).

**Fig. 4:**
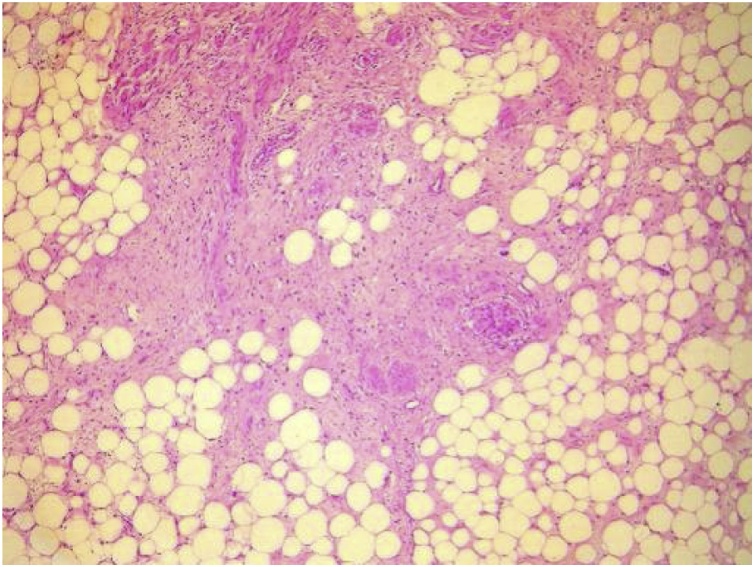
Areas of well-differentiated liposarcoma (HEx40).

The histological examination of the tumor confirmed the diagnosis of dedifferentiated liposarcoma (grade III of FNLCC). Surgical limits were clear (Fig. 3, Fig. 4).

The postoperative course was uneventful. The patient was discharged 10 days after the intervention. After one year of follow up, the patient was complaining of constipation and emerging abdominal swelling. Abdominal and pelvic Computerized Tomography scan showed a recurrent retroperitoneal mass measuring 30 cm compressing the intestines. The mass was completely resected through the same incision. The histological examination confirmed a local recurrence of a liposarcoma. The patient died two days after the procedure due to neurological distress.

## 3 Discussion

Primary retroperitoneal tumors are rare and represent about 0.1−0.2% of all malignant tumors and about 15 % of soft tissue sarcomas in adults [3]. The presence of clinical symptoms at diagnosis such as palpability of the tumor and pain/fullness of the abdomen are associated with a poorer prognosis compared with incidentally diagnosed mass [4].

Liposarcomas are often characterized by an indolent growth in the retroperitoneal space and may cause compression of the adjacent structures or migrate into the scrotum mimicking an inguinoscrotal hernia. In this situation, ‘giant’ liposarcomas over 20 kg are extremely rare. Only a few cases are reported in the literature. Many studies suggested that tumor size impacts the prognosis frequently using a cut-off value of 10 cm [5]. A recent study reported that patients with tumor sized > 15 cm are at high risk of distant recurrence and disease-specific mortality [6].

Liposarcomas are divided into four basic histological categories: myxoid liposarcoma, round cell liposarcoma, well-differentiated liposarcoma, and pleomorphic liposarcoma [7]. The undifferentiated and pleomorphic types are neoplasm with a high grade of malignancy accompanied by remarkable biological aggressiveness and metastatic potential while well-differentiated and myxoid/round cell forms are tumors with a low grade of malignancy, associated with good prognosis [8,9]. The coexistence of multiple histologic subtypes with benign and malignant areas within the same lesion was reported. Such histologic subtypes are usually classified on the basis of the most aggressive cellular components [9].

CT and MRI appearances of liposarcomas can vary according to the combination of these histologic subtypes. CT-Scan and magnetic resonance imaging (MRI) are very useful for defining the size, consistency, and relation between the tumor and the adjacent tissue, and they also allowed us to detect residual tumor and recurrences. The CT imaging of the undifferentiated sarcoma demonstrates a heterogeneous lipogenic mass. In the case of diagnostic doubt and presence of recurrence, Magnetic Resonance Imaging (MRI) could detect with high sensitivity the satellite locations of the main lesion [10].

Surgical treatment aims to achieve negative resection margins, removing any adjacent organs and structures involved. The resection of a retroperitoneal sarcoma of remarkable size is a challenge for the surgeon owing to the anatomical site and the absence of an anatomically evident vascular lymphatic peduncle that makes it hard to obtain safe margin and to the adherences with the contiguous organs. Therefore, the retroperitoneal liposarcoma shows a high rate of local recurrence after surgical excision. Dedifferentiated histologic subtypes and negative surgical margins are associated with poor prognosis [11].

In the literature, 10 %–50 % of retroperitoneal sarcomas are resectable [12,13], and the local recurrence rate ranges from 40 % to 80 % [12,14]. Currently, the overall survival at 5-years reported in the literature for the various histological subtypes well-differentiated, myxoid/round cell, undifferentiated and pleomorphic, ranging from 90 %, 60–90%, 75 %, and 30–50%, respectively [11].

A surgical excision followed by radiotherapy (60–70 Gy) resulted in a significant reduction in local recurrence compared with surgery alone [15]. In another report, the response rate to a doxorubicin and dacarbazine based regimen was 44 % [16], but in the absence of phase II studies, chemotherapy should be considered only for patients with metastatic disease and large, bulky tumors.

An aggressive surgical excision remains the main treatment of retroperitoneal sarcomas, and long-term follow-up examinations are also considered to be important [17]. Tumor extensions from the retroperitoneum should not be confused with primary liposarcomas in the para testicular or inguinal region, which is the preferred site of well-differentiated sclerosing liposarcomas [18].

## 4 Conclusion

Dedifferentiated liposarcomas originated from the retroperitoneum are fully malignant tumors in which identification of dedifferentiated areas is crucial to establish an accurate prognosis. In rare cases, they can extend through the inguinal canal to the scrotum. A surgical resection obtaining negative margins, remains the curative treatment that reduces the risk of recurrence. A careful follow-up to detect early recurrence is essential for optimal care.

## Declaration of Competing Interest

The authors report no declarations of interest.

## Funding

No source to be stated.

## Ethical approval

Ethical approval is not necessary for case report in our locality.

## Consent

Written informed consent was obtained from the patient for publication of this case report and accompanying images. A copy of the written consent is available for review by the Editor-in-Chief of this journal on request.

## Author contribution

Mahjoubi Zied: Writing the manuscript, literature review, final approval of the manuscript and follow up.

Walid Zakhama, Yassine Binous: Surgeons performing the intervention.

Manel Njima: Pathologist performing the histological examination of the tumor.

Walid Zakhama, Aymen Sakly, Aymen Mnasser: Final approval of the manuscript.

Corresponding Author: Mahjoubi Zied.

## Guarantor

Mahjoubi Zied is the Guarantor.

## Provenance and peer review

Not commissioned, externally peer-reviewed.
